# Probiotics Ingestion Does Not Directly Affect Thyroid Hormonal Parameters in Hypothyroid Patients on Levothyroxine Treatment

**DOI:** 10.3389/fendo.2017.00316

**Published:** 2017-11-14

**Authors:** Giorgia Spaggiari, Giulia Brigante, Sara De Vincentis, Umberto Cattini, Laura Roli, Maria Cristina De Santis, Enrica Baraldi, Simonetta Tagliavini, Manuela Varani, Tommaso Trenti, Vincenzo Rochira, Manuela Simoni, Daniele Santi

**Affiliations:** ^1^Unit of Endocrinology, Department of Biomedical, Metabolic and Neural Sciences, University of Modena and Reggio Emilia, Modena, Italy; ^2^Unit of Endocrinology, Department of Medicine, Endocrinology, Metabolism and Geriatrics, Azienda Ospedaliero Universitaria di Modena, Modena, Italy; ^3^Department of Laboratory Medicine and Pathological Anatomy, Azienda USL of Modena, Modena, Italy

**Keywords:** levothyroxine, probiotics, VSL#3^®^, thyroid-stimulating hormone, thyroid hormones, gut microbiota

## Abstract

**Purpose:**

The relationship between probiotics and levothyroxine (LT_4_) requirement has not yet been investigated. The aim of this study was to assess whether a mixture of highly charged *Lactobacilli* and *Bifidobacteria* (VSL#3^®^) is able to influence LT_4_ metabolism acting on the gut microbiota.

**Methods:**

A prospective, randomized, single-blind, controlled, investigator-started clinical trial was carried out. Patients with primary hypothyroidism were randomly assigned to the study (VSL#3^®^ + LT_4_) and the control group (LT_4_). A 2-month treatment phase was followed by 2 months of follow-up. Clinical examination, blood tests for thyroid function and for peripheral tissue markers of thyroid hormones (PTM) were performed monthly. LT_4_ dose adjustments were performed when necessary.

**Results:**

Thirty-nine patients were enrolled in the study group and 41 in the control group. No difference in thyroid function [thyroid-stimulating hormone (TSH), free triiodothyronine (fT_3_), and free thyroxine (fT_4_)] and PTM was found between groups and among visits. FT_3_/fT_4_ ratio was directly correlated to TSH at each visit in both groups, with the exception of the first evaluation of probiotics-treated subjects (rho = 0.287, *p* = 0.076). LT_4_ daily dose adjustments occurred more frequently in the control than in the study group (*p* = 0.007), despite no differences in the mean LT_4_ daily dose. In particular, LT_4_ doses were increased six times in the control group and decreased four times in the study group.

**Conclusion:**

VSL#3^®^ does not directly alter thyroid functional compensation. A probiotics-mediated influence on thyroid hormones homeostasis is suggested since probiotics supplementation could be able to prevent serum hormonal fluctuations.

**ClinicalTrials.gov ID:**

Registration number NCT03095963.

## Introduction

Levothyroxine (LT_4_) is the standard therapeutic choice in hypothyroidism replacement therapy (1). Representing the synthetic equivalent of native tetraiodothyronine (T_4_), LT_4_ modulates the same biological functions (2). In the literature, several peripheral markers have been proposed to measure the peripheral response to thyroid hormones (THs), such as lipid profile [total cholesterol (CH), low density lipoprotein CH, and lipoprotein(a)], coagulation parameters (plasminogen activator inhibitor), hepatic protein synthesis indexes [sex hormone-binding globulin (SHBG) and ferritin], muscular system (creatine phosphokinase and myoglobin), bone metabolism parameters (osteocalcin and urinary N-telopeptide), and selected enzymatic activity [angiotensin-converting enzyme (ACE) and glucose-6-phosphate dehydrogenase] (2).

LT_4_ is administered orally, dissolved by gastric acids and absorbed mainly in jejunum and ileum (1). Many factors influence LT_4_ absorption, such as food and beverages (3–5), drugs (6–13), and pathological conditions impairing the digestive tract absorptive ability (7, 14, 15). Moreover, several drugs are able to interfere with TH requirements increasing LT_4_ metabolism and clearance (4), or acting on expression and activity of catabolic enzymes (16).

The LT_4_ activation and inactivation occur through the action of deiodinases (17). Reversible sulfotransferases and glucuronyltransferases mediate the inactivation of TH in the liver, increasing iodothyronines water-solubility, promoting biliary and urinary clearance (17). In the bowel, gut bacteria expressing β-glucuronidases and sulfatase enzymes are able to hydrolyze glucuronidated and sulfated iodothyronines metabolites (17, 18). In this way, lipophilic deconjugated iodothyronines can be reabsorbed, constituting the enterohepatic recycle of THs (17, 18). According to this mechanism, LT_4_ requirement could be regulated acting on intestinal bacteria and modifying THs enterohepatic recycle. In this context, gut can be considered a possible reservoir of THs (17), although a clear *in vivo* demonstration is still lacking.

Probiotics are defined as live microorganisms which, when consumed in adequate amounts, confer a health effect on the host (19). They are not considered drugs by the current international legislation (19). Probiotics limit the intestinal colonization from pathogenic bacteria (20, 21), improve the physiological bowel barrier function (20, 21), and influence the pro-inflammatory and anti-inflammatory cytokines production (20–23). However, the mechanism by which probiotics contribute to microbiota modulation has not been completely clarified so far (20). Although an effect of probiotics on health has been proposed in several pathologic conditions (18, 20, 24–37), a clear demonstration is still lacking.

Up to now, there is low evidence of probiotics interaction with concomitant drug ingestion, considering their influence on bacteria enzymatic activity (38, 39). Since products containing probiotics are very popular, it would be important to discover any possible interference with pharmacological therapies. This interaction could result in clinical consequences on LT_4_-treated patients simultaneously taking probiotics. This is the first clinical trial designed to investigate whether a mixture of highly charged *Lactobacilli* and *Bifidobacteria* (VSL#3^®^) is able to influence LT_4_ metabolism acting on the gut microbiota in hypothyroid patients.

## Materials and Methods

A prospective, randomized, single-blind, controlled, investigator-started clinical trial was carried out (ClinicalTrials.gov ID: registration number NCT03095963).

Patients’ inclusion criteria were: primary hypothyroidism on LT_4_ replacement therapy, thyroid-stimulating hormone (TSH), fT_3_ and fT_4_ in the normal range, stable LT_4_ dosage during the 6 months before enrolment, Caucasian ethnicity, age 18–65 years. Exclusion criteria were total thyroidectomy for thyroid carcinoma, intestinal malabsorption (e.g., bariatric surgery, inflammatory bowel diseases, celiac disease), ongoing therapies interfering with LT_4_ absorption and/or metabolism (i.e., aluminum-containing antacids, sucralfate, proton pump inhibitors, calcium carbonate, raloxifene, bile acids sequestrants, and ferrous sulfate), and antibiotics treatment in the 6 months before enrolment.

### Study Design

**Figure 1:**
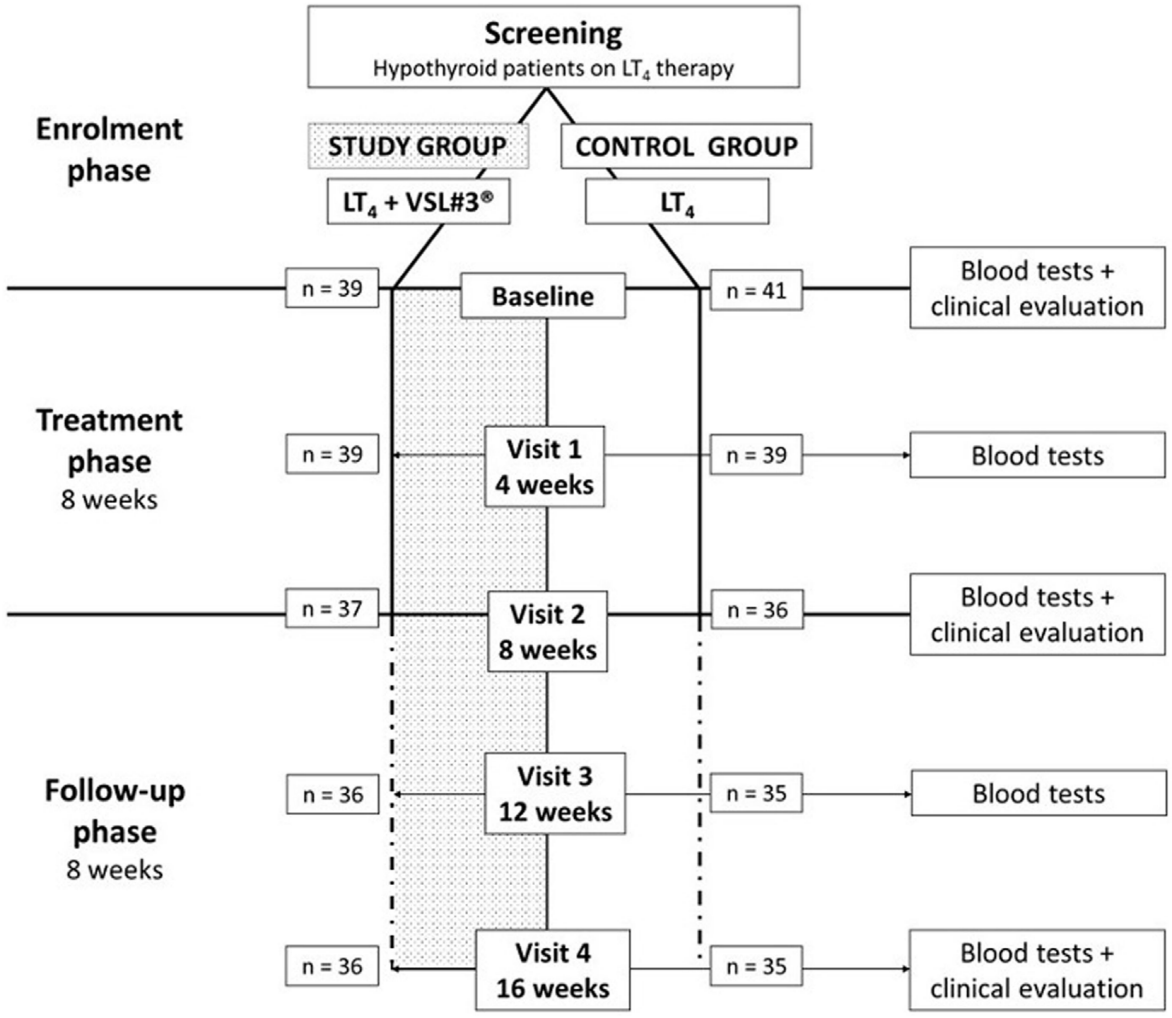
Study design.

Consecutive patients on LT_4_ replacement therapy attending the Endocrinology Unit of Modena (Italy) were screened. According to inclusion and exclusion criteria, 80 participants were enrolled and randomized in study or control group. The random allocation sequence was generated using “Statistical Package for the Social Sciences” software for Macintosh (SPSS) considering a 1:1 ratio by the statistician of the Unit. Clinicians evaluating and enrolling patients were blinded to the randomization list. The study design provided a monthly visit for a 4 months overall time-frame. Patients assigned to the study group took the probiotic supplement VSL#3^®^ for 2 months, followed by a 2-month period of follow-up (Figure 1). All patients were instructed to take LT_4_ tablets with water in the morning with empty stomach, at least 30 min before breakfast and/or other medicine ingestion. Study group patients were taught to assume the probiotic supplement at least 2 h after LT_4_ administration, irrespective of meals, to dissolve it in a cold beverage and to store it in a refrigerator (2–8°C), in order to preserve bacteria load. Patients assigned to the control group continued their LT_4_ therapy without placebo administration.

The study design was single-blind since only the clinician was aware of the allocation. The VSL#3^®^ administration was provided by nurses. Participants were invited to return all used and unused sachets to count the number of opened sachets per the number of treatment days.

All patients underwent five monthly visits (baseline, visit 1, 2, 3, and 4), in which anthropometrical evaluation (weight and height) and hormonal function assessment were performed. A fasting blood sample was taken in the morning at each visit before LT_4_ ingestion. Since TSH results were available the same day of blood sampling, LT_4_ daily dose was promptly adjusted on occurrence of TSH alterations (laboratory reference range: 0.35–4.94 μIU/mL), according to clinical guidelines (40). At baseline, visit 2, and visit 4, patients underwent clinical examination with heart rate and blood pressure evaluation (Figure 1). During each visit, the following data were collected: probiotic ingestion, sex, age, hypothyroidism etiology, LT_4_ formulation dose and brand, body mass index (BMI), body surface area (BSA), systolic blood pressure, diastolic blood pressure, heart rate, TSH, fT_4_, fT_3_, and any possible LT_4_ posology adjustment.

### VSL#3^®^

VSL#3^®^ is a multistrain probiotic supplement available in packets. Each VSL#3^®^ sachet contains a high concentration (450 × 10^9^ CFU) of live freeze-dried bacteria, belonging to eight different strains, i.e., *Bifidobacterium breve, Bifidobacterium longum, Bifidobacterium infantis, Lactobacillus acidophilus, Lactobacillus plantarum, Lactobacillus paracasei, Lactobacillus bulgaricus*, and *Streptococcus thermophilus*. The compound was chosen considering its highest concentration among commonly used probiotics-containing products.

### Laboratory Analyses

During the 4 months evaluation timeline, thyroid function assessment (TSH, fT_3_, fT_4_) was performed monthly to revise LT_4_ dosage if necessary. Blood samples for each patient at each visit were stored at −20°C° until the end of enrolment, to measure peripheral tissue markers of THs. Table S1 in Supplementary Material summarizes methodologies used for each assay and the respective intra- and inter-assay coefficient of variation.

### Statistical Analysis

Considering that no studies so far evaluated the influence of probiotics on hormonal measurements, the sample size was calculated using a previous study aiming at analyzing the influence on LT_4_ absorption. Singh et al. found a TSH decrease of about 70% after concomitant ingestion of calcium and LT_4_ in patients treated for hypothyroidism (41). Thus, assuming a similar variation of TSH in patients treated with both probiotics and LT_4_, using ANOVA univariate statistical analysis, considering a statistical power of 90% and an α-error of 0.05, the required sample size was of 80 patients. Setting a 1:1 case–control ratio, the final sample size was of 40 patients for each group.

Continuous parameters were compared between study and control groups through ANOVA univariate and/or Mann–Whitney test, according to data distribution, evaluated by Kolmogorov–Smirnov test. Categorical variables were compared with Chi-square or Fisher exact test. The changes after treatment were evaluated considering repeated variables, using Wilcoxon signed-rank test. The degree of correlation between ordinal variables was studied using Spearman rho correlation test.

In order to evaluate THs metabolism and pituitary feedback, fT_3_/fT_4_, fT_3_/TSH, and fT_4_/TSH ratios were calculated. Moreover, hormonal measurements were adjusted for the anthropometrical available variables (BMI and BSA).

Statistical analysis was performed using the “Statistical Package for the Social Sciences” software for Macintosh (version 21.0; SPSS Inc., Chicago, IL, USA). Statistical significance was considered significant when *p* < 0.05.

### Ethical Approval

The Institutional Review Board of Modena (Comitato Etico di Modena) approved the study (code 184/13, approved on 12th November 2013).

All procedures performed in studies involving human participants were in accordance with the ethical standards of the institutional and/or national research committee and with the 1964 Helsinki declaration and its later amendments or comparable ethical standards.

### Informed Consent

Written informed consent was obtained from all individual participants included in the study.

## Results

**Figure 2:**
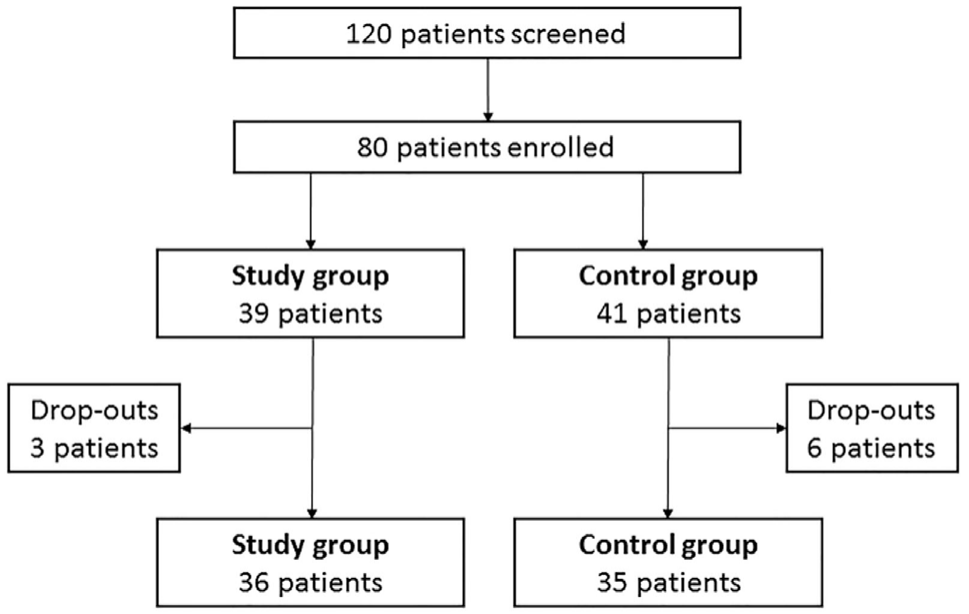
Flow chart of the study.

One hundred and twenty hypothyroid patients on LT_4_ replacement therapy were screened and 80 patients were enrolled according to inclusion and exclusion criteria (Figure 2). Thirty-nine patients (48.8%) entered the study group and 41 (51.2%) the control group. Baseline characteristics of patients are shown in Table 1.

**Table 1:** Patients data and characteristics collected at baseline.

	Reference range	Study group	Control group	*p* Value
Patients number	–	39	41	–
Female, *n* (%)	–	36 (92.3)	41 (100)	0.222
Male, *n* (%)	–	3 (7.7)	0 (0)	0.222
Autoimmune thyroiditis, *n* (%)	–	28 (71.8)	30 (73.2)	0.9160
Non-autoimmune hypothyroidism, *n* (%)	–	11 (28.2)	9 (22)	0.698
Post-thyroidectomy, *n* (%)	–	0 (0)	1 (2.4)	0.960
Post radioiodine, *n* (%)	–	0 (0)	1 (2.4)	0.960
Eutirox^®^, *n* (%)	–	37 (94.9)	37 (90.2)	0.718
Tirosint^®^, *n* (%)	–	1 (2.6)	3 (7.3)	0.644
Non-branded LT_4_, *n* (%)	–	1 (2.6)	1 (2.4)	0.496
Age (years)	–	49.6 ± 10.39	49.33 ± 11.38	0.791
BMI (kg/m^2^)	18.50–24.99	27.77 ± 6.14	27.71 ± 6.12	0.735
SBP (mmHg)	90–120	116 ± 14	115 ± 15	0.703
DBP (mmHg)	60–80	74 ± 8	73 ± 10	0.809
Heart rate (bpm)	60–100	70 ± 6	72 ± 7	***0.043***
TSH (μIU/mL)	0.35–4.94	2.37 ± 1.66	2.72 ± 1.83	0.308
fT_3_ (pg/mL)	1.7–3.7	2.7 ± 0.3	2.6 ± 0.3	0.871
fT_4_ (pg/mL)	7–15	10.8 ± 1.6	10.9 ± 1.6	0.749
LT_4_ dose (μg/day)	–	78.21 ± 26.95	74.56 ± 23.02	0.789
LT_4_ dose (μg/kg/day)	–	1.06 ± 0.38	1.08 ± 0.39	0.855

*Data are expressed as mean ± SD. p value is considered significant when < 0.05*.

*BMI, body mass index; bpm, beats per minute; DBP, diastolic blood pressure; fT_3_, free triiodothyronine; fT_4_, free tetraiodothyronine; LT_4_, levothyroxine; SBP, systolic blood pressure; TSH, thyroid-stimulating hormone*.

At baseline, three male patients (3.75%) entered the study group while no male patients entered the control group. This gender disparity is in line with the known highest female incidence of primary hypothyroidism (1). Three patients (one male and two females) (7.69%) dropped out from the study group and six female patients (14.63%) dropped out from the control group (Figure 2). No adverse events were recorded in both groups and dropouts occurred for patients’ decision.

No differences were observed between study and control group, considering anthropometrical variables (Table 1). Only heart rate was significantly higher in the control than in the study group (*p* = 0.043).

The main cause of hypothyroidism was Hashimoto thyroiditis, occurring with similar incidence both in study and control group (71.8 and 73.2%, respectively; *p* = 0.916). Only two patients (4.88%) in the control group presented an iatrogenic hypothyroidism, due to total thyroidectomy or to radioiodine treatment. The majority of the patients was treated with branded LT_4_ (97.5% in both groups; *p* = 0.971) and only the tablet formulation was used. Among branded LT_4_, Eutirox^®^ was the mostly taken (94.9% in the study vs. 90.2% in the control group; *p* = 0.432). The remaining patients were treated with Tirosint^®^ (2.6% in the study group vs. 7.3% in the control group; *p* = 0.644).

The calculated compliance for probiotics ingestion was 79.2% in the study group.

### Thyroid Hormones

Thyroid-stimulating hormone, fT_3_, fT_4_ did not change between study and control group at each visit, as well as among visits in the two groups (Table 2). The lack of significance was confirmed after adjustment for BMI and BSA or considering hormonal ratios (fT_3_/fT_4_, fT_3_/TSH, and fT_4_/TSH).

**Table 2:** Thyroid axis hormonal measurements.

	Reference range	Study group	Control group	*p* Value
TSH (μIU/mL)	0.35–4.94			
Baseline		2.37 ± 1.66	2.81 ± 1.88	0.308
Visit 1 (treatment phase)		2.35 ± 1.68	2.97 ± 3.38	0.565
Visit 2 (treatment phase)		2.17 ± 1.84	3.88 ± 7.57	0.344
Visit 3 (follow-up phase)		2.53 ± 3.08	2.84 ± 2.47	0.343
Visit 4 (follow-up phase)		2.23 ± 1.72	3.27 ± 4.24	0.344
*p* value	–	0.896	0.970	–

fT_3_ (pg/mL)	1.7–3.7			
Baseline		2.7 ± 0.3	2.6 ± 0.3	0.871
Visit 1 (treatment phase)		2.7 ± 0.4	2.7 ± 0.3	0.894
Visit 2 (treatment phase)		2.7 ± 0.4	2.7 ± 0.3	0.759
Visit 3 (follow-up phase)		2.6 ± 0.4	2.7 ± 0.3	0.079
Visit 4 (follow-up phase)		2.7 ± 0.4	2.7 ± 0.3	0.809
*p* value	–	0.873	0.592	–

fT_4_ (pg/mL)	7–15			
Baseline		10.8 ± 1.6	10.9 ± 1.6	0.902
Visit 1 (treatment phase)		10.9 ± 1.3	11.0 ± 1.6	0.850
Visit 2 (treatment phase)		10.9 ± 1.3	10.9 ± 1.6	0.962
Visit 3 (follow-up phase)		10.7 ± 1.2	10.9 ± 1.6	0.820
Visit 4 (follow-up phase)		10.9 ± 1.6	10.7 ± 1.7	0.424
*p* value	–	0.968	0.805	–

*Data are expressed as mean ± SD. p value is considered significant when < 0.05*.

*fT_3_, free triiodothyronine; fT_4_, free tetraiodothyronine; TSH, thyroid-stimulating hormone*.

In the control group, TSH was inversely related to fT_4_ and fT_3_ (rho = −0.593, *p* < 0.001 and rho = −0.293, *p* = 0.004, respectively) while fT_4_ and fT_3_ were directly related each other (rho = 0.269, *p* = 0.004), as expected according to TH physiology. Unexpectedly, the correlation between TSH and fT_3_ was lost after adjustment for age (*p* = 0.377) and BMI (*p* = 0.286). Similarly, TSH was inversely related to fT_4_ (rho = −0.547, *p* < 0.001) in the study group, but their correlation to fT_3_ was absent (*p* = 0.516 and *p* = 0.462, respectively). However, in both groups, the correlation was lost when each visit was considered separately, suggesting that the low number of cases evaluated in subgroup analyses impaired the statistical power.

Regarding anthropometrical features at baseline, TSH was directly related to BMI (rho = 0.227, *p* = 0.047), but not to BSA (rho = 0.167, *p* = 0.146). Accordingly, fT_4_ was inversely related to BMI (rho = −0.260, *p* = 0.023), while fT_3_ did not correlate with any anthropometrical variable. Moreover, a direct correlation between TSH serum levels and systolic and diastolic pressures was found (rho = 0.282, *p* = 0.012 and rho = 0.227, *p* = 0.046, respectively). On the contrary, neither fT_4_ nor fT_3_ correlated with blood pressure. Similarly to what obtained for hormonal data, these relationships were lost considering following visits. Moreover, heart rate did not correlate to THs at any time point.

Evaluating the peripheral conversion of TH, the fT_3_/fT_4_ ratio was directly related to TSH at each visit in the control group, as expected for the known feedback mechanism. In the study group, this correlation was lost at visit 1 (rho = 0.287, *p* = 0.076), after one month of probiotics treatment, suggesting a possible probiotics effect on this central pathway.

### LT_4_ Dose Adjustments

LT_4_ therapy was adjusted 10 times in 8 patients (10% of the total number of patients). In particular, LT_4_ doses were increased six times in four patients (the same patient needed three dosage adjustments) in the control group and decreased four times in four different patients in the study group (Table 3). Dose adjustments were performed upon occurrence of alterations of serum TSH levels, as suggested by clinical guidelines (40). Interestingly, LT_4_ dose adjustments occurred more frequently in the control than in the study group (*p* = 0.007). However, the mean LT_4_ daily dose did not differ between groups (*p* = 0.419) and among visits (*p* = 0.916 in the control group and *p* = 0.970 in the study group). Considering patients with unchanged LT_4_ dose during the trial (90% of the total number of patients), the lack of hormonal changes after probiotic treatment remained.

**Table 3:** Distribution of LT_4_ dose adjustments among groups.

	LT_4_ dose decrease	LT_4_ dose increase	Total
**All patients**			
Number of patients	4	4	8
Times of LT_4_ dose adjustments	4	6	10
**Study group**			
Number of patients	4	0	4
Times of LT_4_ dose adjustments	4	0	4
**Control group**			
Number of patients	0	4	4
Times of LT_4_ dose adjustments	0	6	6

*LT_4_, levothyroxine*.

As expected, LT_4_ dose was directly related to fT_4_ in the study (rho = 0.299, *p* = 0.001) and control group (rho = 0.457, *p* < 0.001), respectively. Moreover, LT_4_ dose was inversely related to TSH in the study (rho = −0.448, *p* < 0.001) and control group (rho = −0.205, *p* = 0.029), respectively. Considering each visit separately these relationships remained. However, LT_4_ dose was not correlated with serum fT_3_ levels in any group (study group: rho = −0.041, *p* = 0.579; control group: rho = −0.079, *p* = 0.289).

### Peripheral Tissue Markers

All the peripheral tissue markers of THs evaluated did not change between groups or among visits (Table 4).

**Table 4:** Peripheral tissue markers of thyroid hormones.

	Reference range	Study group	Control group	*p* Value
CH (mg/dL)	<200			
Baseline		219 ± 30	222 ± 37	0.626
Visit 1 (treatment phase)		226 ± 40	224 ± 39	0.780
Visit 2 (treatment phase)		213 ± 31	214 ± 48	0.898
Visit 3 (follow-up phase)		217 ± 39	219 ± 40	0.743
Visit 4 (follow-up phase)		220 ± 27	225 ± 45	0.582
*p* value	–	0.496	0.853	–

CK (U/L)	10–171			
Baseline		93 ± 50	116 ± 10	0.658
Visit 1 (treatment phase)		77 ± 40	98 ± 77	0.240
Visit 2 (treatment phase)		77 ± 38	103 ± 75	0.085
Visit 3 (follow-up phase)		115 ± 137	107 ± 106	0.759
Visit 4 (follow-up phase)		81 ± 40	97 ± 86	0.864
*p* value	–	0.636	0.840	–

Myoglobin (ng/mL)	15–106			
Baseline		24 ± 11	25 ± 11	0.616
Visit 1 (treatment phase)		21 ± 8	23 ± 15	0.599
Visit 2 (treatment phase)		21 ± 6	23 ± 8	0.165
Visit 3 (follow-up phase)		23 ± 13	24 ± 10	0.368
Visit 4 (follow-up phase)		23 ± 7	24 ± 9	0.729
*P* value	–	0.569	0.545	–

Ferritin (ng/mL)	25–400			
Baseline		51 ± 61	44 ± 27	0.102
Visit 1 (treatment phase)		47 ± 53	43 ± 31	0.201
Visit 2 (treatment phase)		41 ± 43	47 ± 37	0.078
Visit 3 (follow-up phase)		40 ± 47	41 ± 31	0.222
Visit 4 (follow-up phase)		41 ± 41	41 ± 27	0.202
*P* value	–	0.992	0.881	–

Lipoprotein(a) (mg/dL)	1–30			
Baseline		21 ± 18	22 ± 28	0.516
Visit 1 (treatment phase)		21 ± 21	20 ± 25	0.406
Visit 2 (treatment phase)		19 ± 18	20 ± 25	0.547
Visit 3 (follow-up phase)		19 ± 19	17 ± 19	0.537
Visit 4 (follow-up phase)		22 ± 19	19 ± 25	0.238
*p* value	–	0.967	0.980	–

Osteocalcin (ng/mL)	4.6–65.4			
Baseline		16.0 ± 5.6	16.2 ± 5.2	0.832
Visit 1 (treatment phase)		15.4 ± 5.8	14.4 ± 6.5	0.475
Visit 2 (treatment phase)		15.1 ± 5.7	14.2 ± 7.1	0.557
Visit 3 (follow-up phase)		14.7 ± 6.5	14.7 ± 5.2	0.990
Visit 4 (follow-up phase)		14.4 ± 6.0	14.7 ± 6.2	0.879
*p* value	–	0.826	0.598	–

ACE (U/L)	8–52			
Baseline		35 ± 13	33 ± 15	0.551
Visit 1 (treatment phase)		30 ± 11	31 ± 15	0.755
Visit 2 (treatment phase)		31 ± 12	29 ± 15	0.495
Visit 3 (follow-up phase)		32 ± 13	33 ± 21	0.820
Visit 4 (follow-up phase)		31 ± 12	32 ± 15	0.668
*p* value	–	0.361	0.751	–

SHBG (nmol/L)	Female 19.8–155.2			
	Male 13.5–71.4			
Baseline		78.9 ± 57.2	71.3 ± 41.3	0.981
Visit 1 (treatment phase)		70.5 ± 45.8	69.4 ± 41.4	0.992
Visit 2 (treatment phase)		74.3 ± 52.7	72.04 ± 42.2	0.915
Visit 3 (follow-up phase)		72.62 ± 55.7	72.0 ± 40.0	0.534
Visit 4 (follow-up phase)		78.5 ± 55.0	71.3 ± 37.9	0.806
*p* value	–	0.951	0.994	–

*Data are expressed as mean ± SD. p value is considered significant when < 0.05*.

*ACE, angiotensin-converting enzyme; CH, total cholesterol; CK, creatine kinase; SHBG, sex hormone-binding globulin*.

Considering baseline, TSH was directly related to CH (rho 0.278, *p* < 0.001), CK (rho = 0.143, *p* = 0.007), myoglobin (rho = 0.134, *p* = 0.010), and ferritin (rho = 0.187, *p* < 0.001) and inversely to SHBG (rho = −0.160, *p* = 0.002). FT_4_ was inversely related to CH (rho = −0.247, *p* < 0.001) and myoglobin (rho = −0.112, *p* = 0.033) and directly to SHBG (rho = 0.211, *p* < 0.001). FT_3_ was inversely related to lipoprotein(a) (rho = −0.185, *p* < 0.001) and osteocalcin (rho = −0.105, *p* = 0.045). At visit 2, TSH remained directly related to CH (rho = 0.389, *p* = 0.017) and inversely to SHBG (rho = −0.326, *p* = 0.049). Accordingly, fT_4_ remained inversely related to CH (rho = −0.372, *p* = 0.025) and directly to SHBG (rho = 0.443, *p* = 0.007). These results confirm that CH and SHBG are the most significant tissue effectors of THs action.

Sixty patients were hypercholesterolemic (30 in the study group and 30 in the control group) at baseline, considering the proposed cutoff of CH > 200 mg/dL. Considering only patients with high CH serum levels, peripheral tissue markers did not change among visits and between groups.

### Multivariate Analyses

Stepwise multivariate linear analyses were performed considering TSH, fT_3_, and fT_4_ as dependent variables and anthropometrical parameters as independent. No significant models were generated in any group.

## Discussion

Although the beneficial effects of probiotics were demonstrated in several physiopathological conditions, their impact on thyroid function in humans was not evaluated so far. Here, we found that the intake of a probiotic mixture does not alter directly LT_4_ therapy compensation in patients with primary hypothyroidism on substitutive treatment. Moreover, the probiotics-induced modification of intestinal bacteria seems to be not able to influence LT_4_ absorption when probiotics are administered at least 2 h after LT_4_ intake, since no hormonal serum variation was observed. While serum levels of TSH, fT_3_, and fT_4_ reflect drug absorption, the tissue markers analyzed in this study express THs action at the peripheral level. As a confirmation, no change after probiotics administration was observed. The lack of variation in both circulating hormone levels and THs tissue effect is consistent with the absence of differences in the therapeutic needs between groups and among visits. Nevertheless, a significant difference considering LT_4_ adjustments required in the two groups was found. Interestingly, controls needed significantly higher LT_4_ dose adjustments in comparison with cases, even if no changes in the mean LT_4_ daily dose occurred. We speculate that concomitant probiotics intake could increase the stability in thyroid function compensation. Thus, gut microbiota modification could allow an increased LT_4_ bioavailability, drawing from the THs intestinal reservoir through enterohepatic recycle. Indeed, the iodothyronines deconjugation is mediated by bacterial enzymes sulfatases and β-glucuronidases and the VSL#3^®^ action on gut microbiota could influence the availability of these enzymes. However, in physiological conditions, enterohepatic recycle contributes minimally to circulating THs levels, since sulfated iodothyronines undergo rapid degradation by type 1 deiodinase (17) and deglucuronidation processes seem less important in humans than in animal models (17). This could be the reason of the lack of significant hormonal alterations in this population. However, it is not possible to exclude the presence of a “lessebo effect.” This is described as a negative patients’ expectation, related to the possibility of not receiving the “active” treatment (42). For this reason, control group patients were possibly less motivated to respect LT_4_ administration rules while probiotics-treated patients could have a higher motivation.

Regression analyses confirm the known correlations among THs and BMI. Considering peripheral tissue markers of THs, both TSH and fT_4_ are related with CH and SHBG levels, confirming that these markers are sensible to THs (2). Moreover, the fT_3_/fT_4_ ratio showed a direct correlation to TSH in the control group at each visit, according to physiological feedback mechanism. However, this relationship was lost in the study group after 1 month of probiotic treatment. Thus, probiotics should influence the deiodinases activity, temporarily reducing the feedback. This effect could be explained considering the known anti-inflammatory property of VSL#3^®^, through the decrease in circulating pro-inflammatory cytokines (43). Thus, considering that deiodinases expression and activity can be modulated by cytokines (44–48), probiotics could interfere with THs deiodination. This result reinforces the hypothesis that VSL#3^®^ is able to influence THs metabolism, although a final and significant difference in hormonal levels is not detected. Possibly, a longer treatment might be able to highlight these changes.

Even if VSL#3^®^ is one of the most highly concentrated probiotics largely used in clinical trials, it does not contain strains expressing β-glucuronidases and sulfatases, which could directly increase the enterohepatic recycle of iodothyronines. However, contrasting data are available regarding *Lactobacilli* and *Bifidobacteria* effects on intestinal β-glucuronidase activity (29, 49). Thus, the VSL#3^®^ modulation effect on microbiota by stimulating bacteria growth expressing β-glucuronidases and sulfatases may be only a speculation.

The main limit of this study is represented by the lack of placebo in the control group. It could limit the strength of our results, constituting a sort of “volunteer bias.” Accordingly, the dropout rate is higher in the control than in the study group (six vs. three patients), although not significant. Secondly, the difficulty to evaluate probiotics treatment compliance is another notable limit. The sachets counting expresses an estimation of therapeutic compliance, but differences in microbiota composition and enzymatic activity before and after probiotics ingestion were not evaluated. Moreover, the compliance should be measured considering dietary assessment, which was not recorded in our trial. In the literature, several beneficial effects of VSL#3^®^ were demonstrated, even if no precise evidence about the minimum probiotics concentration required to influence microbiota is available, representing a limit in all trials using probiotics. Furthermore, despite 2 months treatment period seems to be appropriate to modify gut microbiota (50, 51), it is not possible to confirm the exact time-interval needed to obtain a significant change in microbiota composition. Thus, further trials using higher probiotics concentration for longer time should be considered. Finally, in this setting, the accuracy of the TH assay methods remains a crucial point. Commonly used immunoassays could present discrepancies in TH measurement compared to the gold standard, liquid chromatography-tandem mass spectrometry (LC-MSMS), mainly due to pharmacological interferences or pathological conditions able to modify serum transport proteins (52). Nevertheless, the use of LC-MSMS is not currently routinely available in clinical practice and our study population does not present the common conditions that may undermine the immunoassay reliability.

In conclusion, this is the first randomized controlled trial evaluating possible interactions among probiotics, LT_4_ therapy, and pituitary–thyroid axis. Even if these results show no significant differences in hormonal parameters between groups and among visits, a possible probiotics interaction with THs homeostasis is proposed.

These data generate the hypothesis of a “stabilization” of LT_4_ treatment after probiotics ingestion, suggested by the reduced dose adjustments needed in the treatment group, as well as the need of a dose reduction observed only in subjects assuming probiotics. In particular, VSL#3^®^ seems to be able to prevent hormonal fluctuations, perhaps modulating THs enterohepatic recycle and justifying the absence of differences in serum hormonal levels. The effect, however, is limited and no major clinical consequences can be proven at this stage.

The possible probiotics role as “LT_4_ therapy stabilizers” could be an endearing research starting point for both endocrinologists and investigators concerned with probiotics, in order to clarify the interaction between intestinal environment and endocrine system. This association was not evaluated so far and further studies should explore the relationship between gut microbiota and THs, considering other probiotic strains or longer treatment.

## Ethics Statement

The Institutional Review Board of Modena (Comitato Etico di Modena) approved the study (code 184/13, approved on 12th November 2013). All procedures performed in studies involving human participants were in accordance with the ethical standards of the institutional and/or national research committee and with the 1964 Helsinki declaration and its later amendments or comparable ethical standards. Written informed consent was obtained from all individual participants included in the study.

## Author Contributions

GS collected samples, analyzed the data, and wrote the manuscript. GB conceived the study and wrote the manuscript. SV and UC collected clinical and biochemical data. LR, MS, EB, ST, MV, and TT performed laboratory assays. VR contributed writing the manuscript. MS conceived the study and contributed writing the manuscript. DS conceived the study, analyzed the data, and wrote the manuscript. All authors edited the manuscript or revised it critically for important intellectual content and approved the final draft.

## Conflict of Interest Statement

All authors declare that there is no conflict of interest that could be perceived as prejudicing the impartiality of the research reported.
