# Supplementary material for: Probiotics Ingestion Does Not Directly Affect Thyroid Hormonal Parameters in Hypothyroid Patients on Levothyroxine Treatment
Source: Front Endocrinol (Lausanne). 2017 Nov 14;8:316. doi: 10.3389/fendo.2017.00316 (PMC5694461; doi:10.3389/fendo.2017.00316)
Supplement: Supplementary file 1 [file Table_1.DOCX]

**Supplementary table 1**. Methods used for laboratory analyses.

|  | **Reference range** | **Type of assay method** | **Company** | **CV intra-assay** | **CV inter-assay** |
| --- | --- | --- | --- | --- | --- |
| **TSH**  (µIU/mL) | 0.35-4.94 | chemiluminescent microparticle immunoassay | Abbott Diagnostics, USA | 3.10% | 3.50% |
| **fT_3_**  (pg/mL) | 1.7-3.7 | chemiluminescent microparticle immunoassay | Abbott Diagnostics, USA | 2.80% | 3.65% |
| **fT_4_**  (pg/mL) | 7-15 | chemiluminescent microparticle immunoassay | Abbott Diagnostics, USA | 3.80% | 5.70% |
| **CH**  (mg/dL) | <200 | enzymatic colorimetric assay | Beckman Coulter Inc, USA | 0.81% | 1.25% |
| **CK**  (U/L) | 10-171 | UV Kinetic test | Beckman Coulter Inc, USA | 1.50% | 3.87% |
| **Myoglobin** (ng/mL) | 15-106 | chemiluminescent immunoassay Access | Beckman Coulter Inc, USA | 1.89% | 3.29% |
| **Ferritin** (ng/mL) | 25-400 | chemiluminescent immunoassay Access | Beckman Coulter Inc, USA | 3.25% | 5.2% |
| **Lp(a)** (mg/dL) | 1-30 | immunoturbidimetric quantitative immunoassay | Sentinel Diagnostics, Italy | 1.63% | 1.63% |
| **Osteocalcin** (ng/mL) | 4.6-65.4 | chemiluminescent immunoassay Liaison XL | DiaSorin, Italy | 5.00% | 5.00% |
| **ACE**  (U/L) | 8-52 | Kinetic test | Trinity Biotech Plc, Ireland | 2.33% | 4.33% |
| **SHBG**  (nmol/L) | Female 19.8-155.2  Male  13.5-71.4 | chemiluminescent immunoassay Architect | Abbott GmbH & Co, Germany | 10.00% | 10.00% |

[ACE = angiotensin-converting enzyme; CH = total cholesterol; CK = creatine kinase; CV = coefficient of variation; fT_3_ = free triiodothyronine; fT_4_ = free tetraiodothyronine; Lp(a) = lipoprotein(a); TSH = thyroid-stimulating hormone; SHBG = sex hormone binding globulin].
